# Metabolic features of myeloma cells in the context of bone microenvironment: Implication for the pathophysiology and clinic of myeloma bone disease

**DOI:** 10.3389/fonc.2022.1015402

**Published:** 2022-10-13

**Authors:** Vincenzo Raimondi, Denise Toscani, Valentina Marchica, Jessica Burroughs-Garcia, Paola Storti, Nicola Giuliani

**Affiliations:** ^1^ Department of Medicine and Surgery, University of Parma, Parma, Italy; ^2^ Hematology, “Azienda Ospedaliero-Universitaria di Parma”, Parma, Italy

**Keywords:** metabolism, osteoblast, osteoclast, myeloma, glutamine, imaging

## Abstract

Multiple myeloma (MM) is a hematological malignancy characterized by the accumulation of malignant plasma cells (PCs) into the bone marrow (BM). The complex interaction between the BM microenvironment and MM PCs can lead to severe impairment of bone remodeling. Indeed, the BM microenvironment exerts a critical role in the survival of malignant PCs. Growing evidence indicates that MM cells have several metabolic features including enhanced glycolysis and an increase in lactate production through the upregulation of glucose transporters and enzymes. More recently, it has been reported that MM cells arehighly glutamine addicted. Interestingly, these metabolic changes in MM cells may affect BM microenvironment cells by altering the differentiation process of osteoblasts from mesenchymal stromal cells. The identification of glutamine metabolism alterations in MM cells and bone microenvironment may provide a rationale to design new therapeutic approaches and diagnostic tools. The osteolytic lesions are the most frequent clinical features in MM patients, often characterized by pathological fractures and acute pain. The use of the newer imaging techniques such as Magnetic Resonance Imaging (MRI) and combined Positron Emission Tomography (PET) and Computerized Tomography (CT) has been introduced into clinical practice to better define the skeletal involvement. Currently, the PET/CT with ^18^F-fluorodeoxyglucose (FDG) is the diagnostic gold standard to detect active MM bone disease due to the high glycolytic activity of MM cells. However, new tracers are actively under investigation because a portion of MM patients remains negative at the skeletal level by ^18^F-FDG. In this review, we will summarize the existing knowledge on the metabolic alterations of MM cells considering their impact on the BM microenvironment cells and particularly in the subsequent formation of osteolytic bone lesions. Based on this, we will discuss the identification of possible new druggable targets and the use of novel metabolic targets for PET imaging in the detection of skeletal lesions, in the staging and treatment response of MM patients.

## Introduction

Multiple myeloma (MM) is a malignant plasma cells (PCs) disorder characterized by infiltration of clonal PCs in the bone marrow (BM) and the production of monoclonal immunoglobulin, leading to end-organ damage (1).

It has been well established that metabolic reprogramming is considered one of the main features of tumor cells needed to maintain their malignant phenotype (2). Glucose and glutamine are the two principal nutrients employed by cancer cells to fulfill their biosynthetic demands (3). Moreover, the critical role of the tumor microenvironment has long been recognized in cancer biology, including metabolic reprogramming (4). Consistently, in MM it has been reported that malignant PCs experience metabolic changes compared to their healthy PCs counterparts (5–7). Moreover, recent data indicate that MM cells are typically characterized by glutamine (Gln) addicted feature that alters the physiological Gln levels in the BM microenvironment with a significant impact also on bone remodeling (7).

As known, osteoblasts (OBs) and osteoclast (OCs) are specialized cells responsible for bone formation and resorption process, respectively. The pathogenesis of osteolytic bone lesions in MM involves uncoupling of the bone remodeling processes. Altered bone remodeling is characterized by an increased osteoclastic formation and a reduced osteoblastic formation (8, 9). Bone remodeling requires a significant amount of energy (1). On this basis, it is not surprising that alterations in the energy metabolism of bone cells caused by the different availability of metabolites in the MM microenvironment alter their differentiation and function (7, 10). However, at present, our understanding of how the metabolic interaction between malignant PCs and the bone microenvironment steers the progression of the disease is limited.

The osteolytic bone lesions are the most frequent clinical features present in MM patients. In fact, MM bone disease greatly affects the patient’s quality of life, with a high morbidity and mortality rate (11, 12). The evaluation of the presence of bone disease is undoubtedly essential for the diagnosis and staging of MM patients. Many imaging techniques have been proposed to detect the skeletal involvement of MM. Given the multiple options available for the detection of bone lesions, the International Myeloma Working Group (IMWG) recently established guidelines on the optimal use of imaging methods at different disease stages (13). Based on the IMWG recommendations, whole-body Computed Tomography (CT) scan is the first-choice imaging technique to identify and assess the presence and the extent of osteolytic lesions (13). In addition, if whole-body CT is negative, and no other myeloma-defining events are present, the IMWG recommends the use of whole-body Magnetic Resonance Imaging (MRI) to exclude the presence of focal lesions (13). Another technique of preference is the Positron Emission Tomography (PET)/CT which draws on information from the metabolic activity of tumor cells within the investigated area that uptake a radioactive tracer (14). Given the high glycolytic activity of MM cells, which are characterized by up-regulation of transporters and enzymes responsible for glucose metabolism, the most widely used PET tracer is ^18^F-fluorodeoxyglucose (^18^F-FDG) (15). The PET/CT can be used in place of whole-body CT or whole-body MRI to detect bone lesions in MM patients with a prognostic value especially in young MM patients eligible for high dose therapy (13). Nevertheless, ^18^F-FDG PET/CT is associated with several limitations and for this reasons, alternative tracers have been investigated.

In this review, we will discuss the metabolic features of MM cells and how these alterations impair OBs-OCs differentiation within the bone microenvironment. Finally, we explore the possible use of new PET tracers that can help to overcome the current limitations of the standard tracer, the ^18^F-FDG.

## Metabolic reprogramming of cancer cells

It is well established that cancer cells produce a large amount of lactate even under the presence of optimal oxygen concentration. This effect called “The Warburg effect” was firstly described by Otto Warburg in the 1920s. He showed that tumor cells increase their consumption of glucose coupled with an increased lactate production regardless of the available oxygen level (16, 17). Warburg concluded that cancer cells have an impairment in mitochondrial activity and subsequent dependence on glycolysis. Later studies demonstrated that most cancer cells are not characterized by mitochondrial dysfunction (18, 19) suggesting a different explanation for aerobic glycolysis. So why do cancer cells shift their metabolism toward a less efficient mechanism of energy generation? The existence of this metabolic reprogramming is necessary for cancer cells to obtain the energy required for maintaining their high proliferative state. For this purpose, cells need to generate energy quickly. As stated above, the generation of ATP through glycolysis is less efficient but faster than aerobic respiration. Moreover, glucose, the most abundant extracellular nutrient, provides cells with the molecules necessary to sustain biosynthetic pathways (2). Additionally, the high lactate production generates an acidic microenvironment where only cancer cells can grow. As intensively reviewed by others, cancer cells upregulate glucose transporters, such as GLUT1, GLUT2, GLUT3, and GLUT4 to increase their glucose uptake (20). This specific characteristic of cancer cells has been widely exploited in PET imaging using ^18^F-FDG tracers to visualize the tumors and will be discussed later in the review.

Additionally, oncogenes such as RAS, c-MYC, and HIF-1α are reported to induce glycolysis in cancer (2, 21). In particular, c-MYC and HIF-1α promote the expression of glycolytic enzymes hexokinase 2 (HK2), phosphofructokinase 1 (PFK1), triosephosphate isomerase 1 (TPI1), lactate dehydrogenase A (LDHA) in cancer (22). In contrast, the tumor suppressor gene TP53, largely mutated in different types of cancer, directly impairs glycolysis by downregulating GLUT1, GLUT4, and HK2 and favors oxidative phosphorylation (OXPHOS) (23). However, the theory of “metabolic plasticity” of cancer cells has taken hold in recent years. According to this theory, in tumor cells the OXPHOS is functional and the cells can switch between both, the OXPHOS and the aerobic glycolysis, or even perform them simultaneously (24). This metabolic characteristic confers to the neoplastic cells the ability to adapt to changes in the microenvironments and provides a mechanism for chemoresistance (25). Several studies have shown that the repression of OXPHOS is not required to promote cell growth in various cancer types (e.g., leukemia, a subset of lymphomas, melanoma, and ductal adenocarcinoma of the pancreas) (26–29). Rather, an increase in OXPHOS is present in these cases. The accumulating evidence of the “metabolic plasticity” has led to a shift from the Warburg effect to the “Reverse Warburg Effect”. In this later effect, the stromal cells in the microenvironment are induced by neoplastic cells to undergo aerobic glycolysis and then transfer the products to neoplastic cells for utilization for mitochondrial OXPHOS (30, 31). This cellular metabolic interaction of stromal and neoplastic cells allows tumors to respond to fluctuations in nutrient availability to maximize cellular proliferation and growth (32).

In summary, while aerobic glycolysis has long been considered the dominant metabolic phenotype in cancer, the critical role of OXPHOS in tumorigenesis has recently emerged. This metabolic plasticity allows cancer cells to regulate their metabolic phenotypes to adapt to the microenvironment. Based on these recent findings, it may be necessary to target both, OXPHOS and glycolysis to suppress tumor aggressiveness.

## Glucose metabolism in MM

MM cells are characterized by increased aerobic glycolysis, resulting in elevated lactic acid levels regardless of oxygen availability (15). Consistently, MM cells show a high sensitivity to several glycolytic inhibitors, including dichloroacetate (33).

Multiple factors are involved in the increased glycolysis observed in MM cells. One of the main regulators of cellular metabolic processes, the PI3K-Akt signaling pathway, is upregulated in MM (34). Its dysregulation, induces the expression of several glycolytic enzymes, including HK2 and PFK1, and promotes the translocation and activation of GLUT1 and GLUT4 (35, 36). In particular, GLUT4 has been proved to be the key transporter in sustaining glucose metabolism of MM cells (36). It is possible to act on GLUT4 by administering the HIV protease inhibitor ritonavir, which has an off-target effect on the transporter. *In vitro* and *in vivo* studies using MM cell have shown that treatment with ritonavir results in a reduction of cell proliferation, cell viability, and a concomitant increase in chemosensitivity (36, 37). In contrast to GLUT4, the other transporters such as GLUT1, GLUT8, and GLUT11 have marginal roles in MM cells regarding glucose uptake and lactate extrusion (36).

The peroxisome proliferator-activated receptor gamma co-activator 1 (PGC-1) family plays an important role in the metabolic reprogramming of malignant PCs. The PGC-1α acts as a transcriptional co-activator, regulating several genes implicated in energy metabolism and particularly in the biogenesis of the mitochondria (38). *In vitro* experiments have shown that the inhibition of PGC-1α in MM cells causes a decrease in GLUT4 expression, leading to a reduction in lactate production (39). The other member of the PGC-1 family, the PGC-1β, promotes glycolysis and proliferation of MM cells through increased expression of LDHA (40). Overexpression of PGC-1β in MM cells significantly enhances glycolysis metabolism, whereas the knockdown suppresses glycolysis metabolism with decreased proliferation and tumor growth (40).

The transcription factors FOXM1, HIF-1α and c-MYC also play a critical role during metabolic reprogramming of MM cells. The transcription factor forkhead box M1 (FOXM1) has been identified as a positive regulator of metabolism in MM (41). Cheng et al. demonstrated that FOXM1 increases glucose uptake, lactate production, and oxygen consumption, promoting MM growth and survival (41). Treatment with 1,1-diarylethylene (NB73), a small FOXM1 inhibitory compound, suppresses MM *in vitro* and *in vivo* by enhancing the proteasomal degradation of FOXM1 (41).

It has been demonstrated that HIF-1α induces transcription of several genes that regulate glycolytic enzymes and lactate production in MM cells, including GLUT1, HK2 and LDHA (42).

A relationship between the c-MYC oncogene and the metabolic features of MM cells has been hypothesized (43). c-MYC promotes the expression of glucose transporters and key glycolysis rate-limiting enzymes (44). Specifically, c-MYC induces high expression of pyruvate kinase (PK) M2 (PKM2) (an isoform of PK) in MM cells, through the never-in mitosis (NIMA) related kinase 2 (NEK2), which governs chromosome segregation in the G2/M phase of the cell cycle (45). Indeed, the knockdown of PKM2 results in a reduction of MM cell growth and cell cycle arrest at the G1/S transition (46).

Recently, it has been shown that MM cells not only produce an increased amount of lactate, but also incorporate it through the monocarboxylate transporter 1 (MCT1) in order to generate more energy (47). Moreover, MCT1 MM cells knockdown induces apoptosis through a decrease in lactate influx and ATP production (48). Interestingly, the evidence of lactate incorporation in MM cells suggests its presence in the BM microenvironment. In fact, MM cells are supplied with lactate from the surrounding environment, exploiting the above described Reverse Warburg Effect (48).

Finally, glucose transport and metabolism may be a target of the anti-MM drugs currently in use. It has been reported that treatment of MM cells with vincristine or the proteasome inhibitor bortezomib reduces the expression of GLUT1 and HK2 and induces them to apoptosis (49)

## Gln metabolism in MM cells

Recently, we demonstrated that several human myeloma cell lines (HMCLs) markedly increased ammonium output in presence of Gln and that, consistently, primary BM CD138^+^ PCs from MM patients show a higher level of ammonium output than the BM CD138^-^ cell fraction from the same patient. Moreover, MM patients showed a significantly higher ammonium level in BM plasma than Monoclonal Gammopathy of Undetermined Significance (MGUS) and Smoldering Myeloma (SMM) patients (6). Furthermore, we also showed that these malignant PCs lack Gln synthetase (GS) and consequently rely on extracellular uptake of Gln (6). *In vitro* experiments showed that MM cells undergo cell death when incubated in the absence of Gln and when the extracellular amino acid is deprived by L-asparaginase, which hydrolyzes Gln and asparagine. Furthermore, synergistic effects occur when L-asparaginase is combined with bortezomib or another proteasome inhibitor, carfilzomib, leading to increased cytotoxic effects in MM cells (6, 50, 51). The expression of enzymes involved in Gln metabolism and Gln transporters is also consistent with the strong Gln dependence of MM cells. Both HMCLs and primary CD138^+^ PCs express high levels of kidney-type glutaminase (GLS) while lacking significant expression of GS (6). In addition, MM cells express three key Gln transporters, namely ASCT2, LAT1, and SNAT1, the expression of which gradually increases during disease progression from MGUS to MM. Most of the Gln influx into MM cells occurs through the ASCT2 transporter, whereas the contributions of SNAT1 and LAT1 appear to occur to a minor extent (6).

The transcriptional activity of c-MYC protein is upregulated during the late stages of MM progression and is correlated with poor survival (52). c-MYC is involved in the modulation of both glycolysis, as described above, and glutaminolysis (44). Indeed, c-MYC regulates the transcriptional program involved in glutaminolysis by inducing ASCT2 and GLS (53).

The Gln dependence observed in MM cells provides the rationale for its use both as a therapeutic target (50) and for imaging in PET scans (54).

### Gln transporters as attractive therapeutics targets

The alterations of Gln metabolism have been exploited as a therapeutic strategy to decrease relapse and improve therapy outcome. L-γ-glutamyl-p-nitroanilide (GPNA), the most used ASCT2 inhibitor, significantly reduces Gln uptake and hampers proliferation of MM cells, demonstrating their dependence on extracellular Gln (6). Prelowska et al. reported that ASCT2 inhibitors synergistically enhance the cytotoxic efficacy of carfilzomib by causing programmed cell death and regulating autophagy. Indeed, treatment with ASCT2 and carfilzomib increases intracellular reactive oxygen species (ROS) concentrations and stimulates the unfolded protein response (55). Importantly, knockdown of ASCT2 in MM cells partially reduced cell proliferation *in vitro* and delays the growth of human myeloma xenografts in mouse models (6). Thus, among the different approaches to targeting the transport or metabolism of Gln in MM cells, interference with ASCT2 expression and/or function has been demonstrated to have an effect *in vivo*.

On the other hand, although GLS1-dependent anaplerosis is evident in MM cell lines, the GLS1 inhibitors bis-2-(5-phenylacetamide-1,3,4-thiadiazol-2-yl) ethyl sulfide (BPTES) or telaglenastat (CB-839) were not fully successful when used alone (6). Furthermore, the use of CB-839 in combination with both, bortezomib and pomalidomide results in a significant anti-MM effect. Several clinical trials with CB-839 are currently ongoing (56). MM cell lines resistant to GLS1 inhibitors were very sensitive to Gln starvation (6). These different effects could be explained by considering that the loss of Gln transport from the extracellular compartment limits the availability of the amino acid for all metabolic pathways for which Gln is required. In fact, in addition to its role in protein assembling, Gln is an important nitrogen donor for nucleotide synthesis and the major source of α-ketoglutarate (α-KG) in the tricarboxylic acid (TCA) cycle. Furthermore, it is utilized in the biosynthesis of all non-essential amino acids (57) and its intermediate glutamate acts as an exchange factor for the import of essential amino acids (58).

Gln deprivation has been investigated as an alternative strategy in combination with drugs currently used in the treatment of MM. Several studies have reported that in MM cells, Gln deficiency synergizes with the chemotherapeutic agent bortezomib and venetoclax, a BH3-mimetic drug currently being studied in MM patients, given the rationale toward future combination therapies in MM patients (6, 59).

## Metabolism in the bone microenvironment cells

### Energy metabolism of the OB

OBs are cells differentiated from mesenchymal stem cell progenitors during bone remodeling (60). Throughout differentiation, OBs produced a considerable amount of extracellular matrix proteins (61). To perform this highly energetic process, OBs utilize glycolysis as the main source of energy. Over the years, several studies have demonstrated that OBs have a significantly high uptake of glucose. The use of radiolabeled glucose analogs has confirmed the high uptake of glucose by mouse bone tissue (62). It has also been shown that GLUT transporters are responsible for glucose uptake in OB cells (63). Gene expression analysis in osteoblastic cell lines has shown the expression of GLUT1, GLUT3, and GLUT4 (64, 65). In particular, GLUT1 acts as a transporter in primary OB cells and as a modulator of the post-translational modification of runt-related transcription factor 2 (RUNX2) by suppressing adenosine 5’-monophosphate kinase and blocking RUNX2 ubiquitination (64), while ablation of GLUT4 in primary cultures of mouse OBs suppresses insulin-stimulated glucose uptake, reduces proliferation, and decreases OB maturation measures.

To obtain energy, OBs carry out aerobic glycolysis, even in the presence of high oxygen concentrations, converting glucose into lactate (recalling the Warburg effect) (66). It has also been shown that forcing the activation of glycolysis by overexpressing HIF-1α induces an increase in the number of OBs and bone mass in mice (67). As a result, aerobic glycolysis can be seen not only as a metabolic feature of OBs but also as a component of their phenotype. In addition, it allows to obtain the metabolic intermediates involved in the synthesis of matrix proteins (68). In OBs, aerobic glycolysis may be accompanied by citrate secretion, which is relevant for the formation of apatite nanocrystals in bone (69).

**Figure 1:**
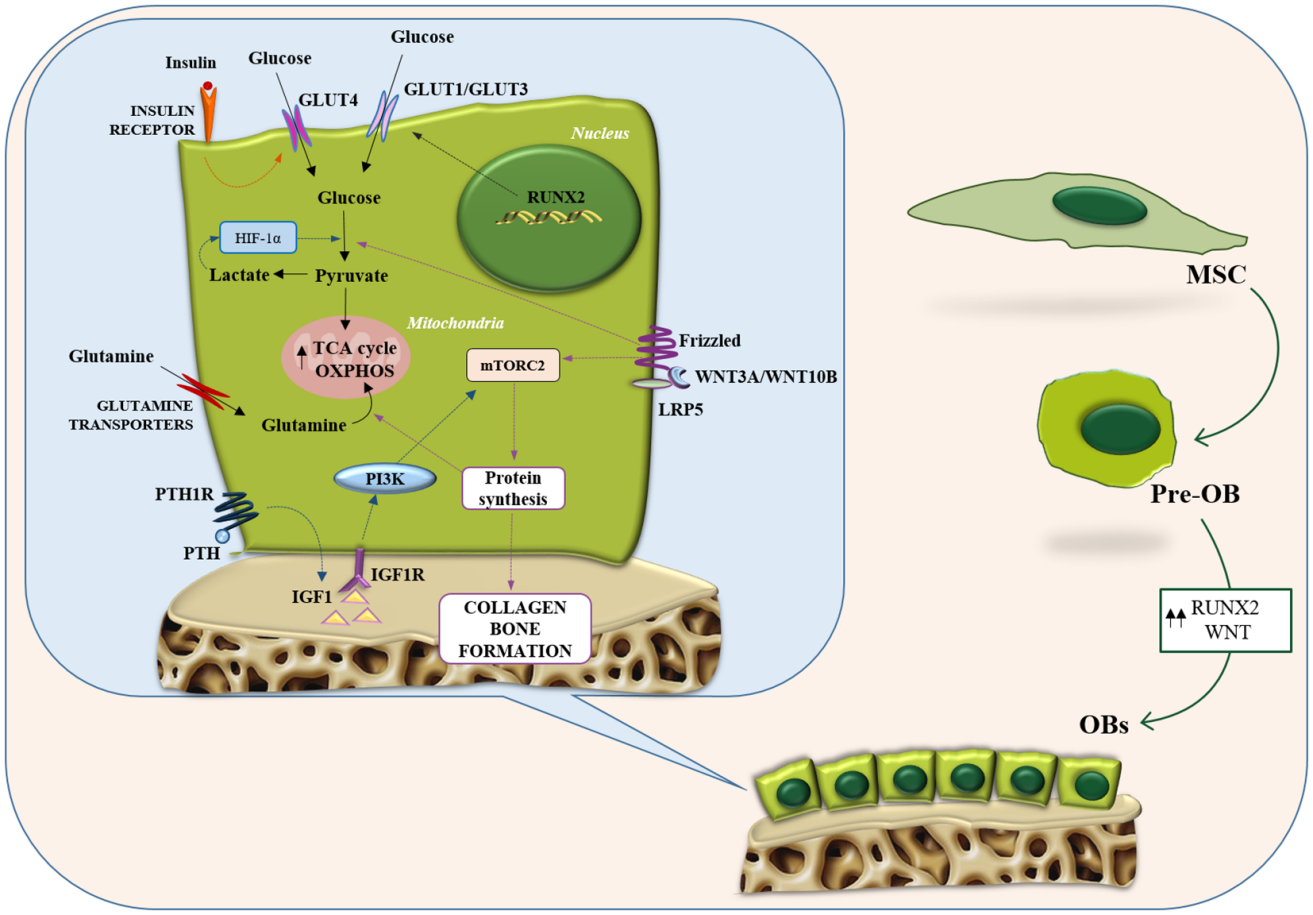
Metabolic features of osteoblasts. Representation of the main metabolic pathways in osteoblasts: glycolysis and glutamine metabolism. Intracellular energy metabolism relies on different feed-forward and negative feedback mechanisms. Osteoblasts take advantage of glucose metabolism to generate ATP through aerobic/anaerobic glycolysis or OXPHOS. Similarly, glutamine metabolism fuels the TCA cycle and boosts the OXPHOS pathway to generate additional ATP. GLUT, glucose transporter; HIF1-α, hypoxia-inducible factor-alpha; IGF-1, insulin-like growth factor-1; IGF1R, insulin like growth factor 1 receptor; LRP5, LDL receptor related protein 5; MSC, mesenchymal stromal cells; mTORC2, mTOR complex 2; OB, osteoblast; OXPHOS, oxidative phosphorylation; PI3K, phosphatidylInositol 3-kinase; PTH, parathyroid hormone; PTH1R, parathyroid hormone 1 receptor; RUNX2, runt-related transcription factor 2; TCA, tricarboxylic acid.

One of the main promoters of OB formation is Wnt signaling. Wnt proteins reprogram cellular glucose metabolism during OBs differentiation (70). Wnt3a, 7b, and 10b stimulate aerobic glycolysis either in cell lines or primary cultures of osteogenic cells. Wnt3a increases the protein levels of several glycolytic enzymes downstream of mTORC2 and Akt (70). Similarly, the anabolic hormone PTH stimulates aerobic glycolysis in long bone or calvarial bone explants and isolated cranial OBs (71, 72) and induces glucose uptake in a rat OB cell line (65). Specifically, to stimulate aerobic glycolysis in pre-osteoblastic MC3T3-E1 cells, PTH activates insulin-like growth factor (IGF) signaling, which triggers the PI3K/mTORC2 and upregulates metabolic enzymes such as HK2, LDH, and pyruvate dehydrogenase kinase 1 (PDK1) (73). Furthermore, it has been shown that deletion of IGF1 or IGF1R in OBs is able to inhibit the bone anabolic effect of PTH in mice (74) (**Figure 1**).

#### Role of Gln in OB formation and function

Different studies have demonstrated that Gln is involved in OB differentiation from BM stromal cells (BMSCs) (75) and is critical for the formation of the matrix mineralization process (7, 76). In OBs precursors, Gln, through an oxidation process at the level of the TCA cycle, is converted into citrate and used to produce energy at the mitochondrial level (77). Experimental evidence suggested GLS and the concentrative Gln transporter sodium-dependent neutral amino acid transporter 2 (SNAT2) are the targeted enzymes of Gln metabolism affecting the differentiation of BMSCs (7). Inhibiting Gln metabolism through deletion of GLS in BMSCs resulted in a reduction of OBs number and the capability of bone formation, consequently causing a decreased bone mass in mice (75). Recent evidence reported that miRNA-206 participates in the bioenergetics of BMSCs by directly bounding to the 3-untranslated region of GLS mRNA suppressing GLS expression and Gln metabolism. This results in the inhibition of osteogenic differentiation of BMSCs (78). Furthermore, the fact that Gln participates in the production of glutathione plays a crucial role in OB precursors survival, as the latter can be exploited to offset the activity of ROS harmful to maturing OBs.

Wnt signaling has been associated with an increased Gln metabolism in OBs (77). Indeed, two amino acid transporters LAT1 and ASCT2 have been identified as the main Gln transporters in response to Wnt activation (79). LAT1 mediates the increase of Gln uptake in response to Wnt activation whereas ASCT2 is involved, in general, in Gln uptake (79).

Furthermore, Gln metabolism plays a vital role in maintaining high levels of aspartate and glutamate in skeletal stem cells, as it contributes both carbon and nitrogen for their biosynthesis (75) (**Figure 1**). Regarding glutamate itself, *in vitro* studies on its role in OBs function have generally focused on the use of N-methyl-D-aspartate (NMDA) glutamate receptor antagonists (such as dizocilpine also known as MK-801). Short-term treatment with both, MK-801 and a glycine receptor domain antagonist, depresses alkaline phosphatase expression by OBs without affecting cell survival (80). Sustained treatment downregulated RUNX2 expression suggesting a role for glutamate in the differentiation regulation. Subsequent investigations showed that NMDA and α-amino-3-hydroxy-5-methyl-4-isoxazole propionic acid (AMPA) glutamate receptor antagonists inhibited osteoblastic differentiation processes in rat BM cultures, leading to the specific observation that the latter became less osteogenic and more adipocytic (81).

### Metabolic features of OC

OCs are multinucleated giant cells formed by the fusion of macrophage family progenitors, which are in charge of bone resorption. Macrophage colony-stimulating factor (M-CSF) and the receptor activator of nuclear factor κB ligand (RANKL) are the two factors necessary to promote osteoclastogenesis (82). OCs appear accommodated in pits excavated on the surface of the trabecular bone, termed Howship lacunae, which are formed precisely by their erosive action (83). Bone matrix resorption begins with the dissolution of the mineral component, due to acidification of the microenvironment; at the same time, proteolytic enzymes, released from the OC, are activated and digest the organic components of the bone matrix (83, 84). Since bone resorption is an energetically wasteful process, it has been suggested that OCs undergo metabolic adaptation during differentiation to accommodate their increased ATP demand. Differentiation of OCs is accompanied by an increased number of mitochondria (85), increased oxygen consumption rate, and an upregulated expression of glycolytic enzymes (e.g., HK, PFK, and PK) (86), TCA cycle, and OXPHOS indicating increased energy production (87). Several factors related to biogenesis and mitochondrial functions including, PGC-1β, peroxisome proliferator-activated receptor y (PPARy), and estrogen-related receptor α (ERRα), play key roles in OC differentiation and function (88–90). OXPHOS is the main bioenergetic source for OC formation (91). Treatment with electron transport chain inhibitors blocks osteoclastogenesis, and cells lacking mitochondrial complex I subunits fail to differentiate into OCs (92). The RANKL-induced increase in mitochondrial respiration of OCs is dependent on MYC/ERRα, suggesting that MYC is a central regulator for their metabolic reprogramming (93). OCs-specific MYC-deficient mice show increased bone mass caused by faulty OCs development, and the bone loss induced by osteoporosis is reduced in MYC-deficient mice (93). Therefore, alteration of OXPHOS in OCs results in changes in bone phenotype by reducing their numbers. On the other hand, OCs bone resorption is enhanced when OXPHOS is low as demonstrated by the treatment with rotenone, an inhibitor of mitochondrial complex I, that increases OC activity (91).

**Figure 2:**
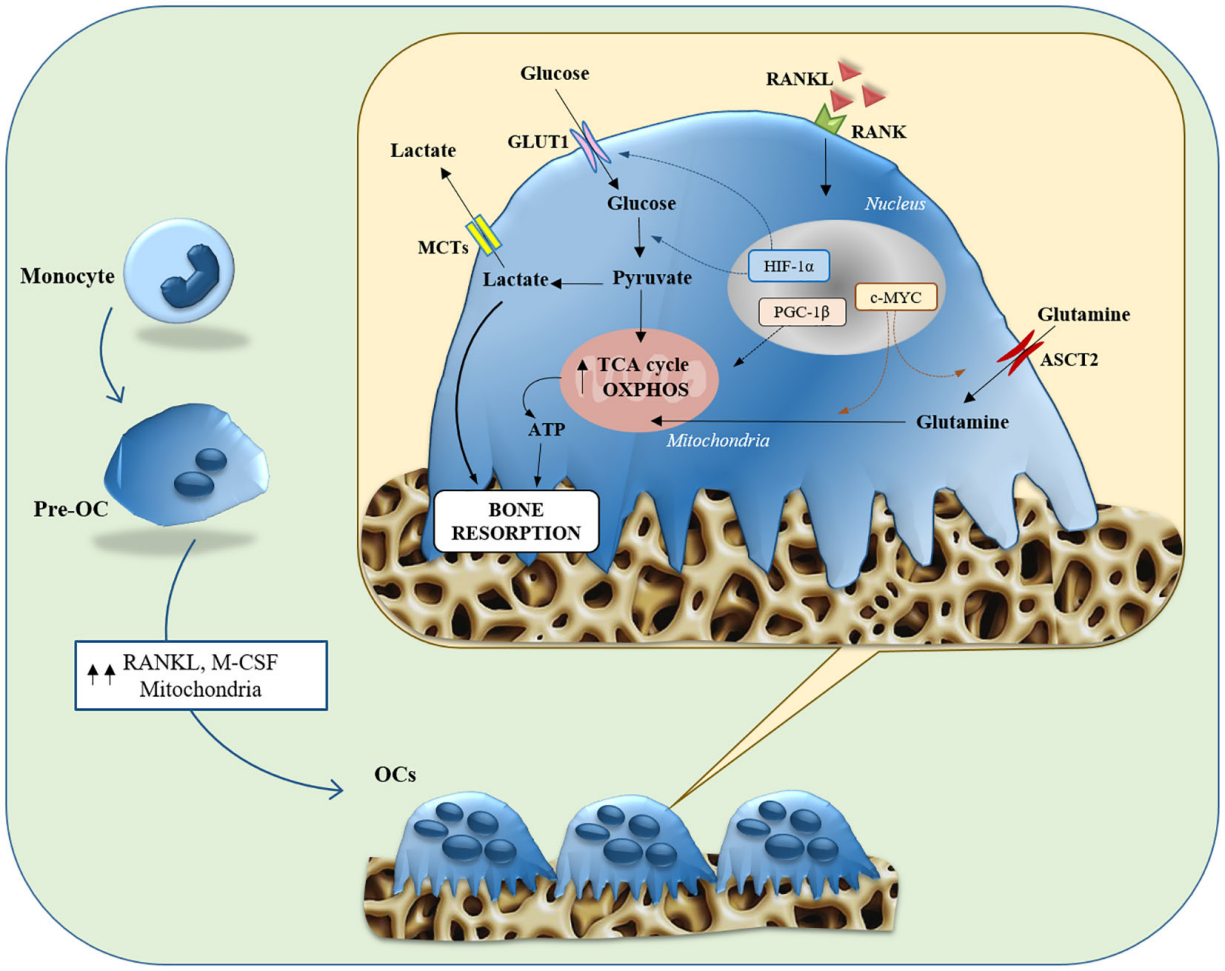
Glucose and glutamine metabolisms in Osteoclasts. Illustration summarizing both glycolysis and glutaminolysis in osteoclast differentiation and bone resorption function. Osteoclasts contain abundant mitochondria, whose biogenesis is stimulated by PGC-1β, due to the bioenergetic demands of osteoclastogenesis and bone resorption. The expression of Glut1 and glycolytic genes is stimulated toward the maturation stage, through the action of HIF-1α, contributing to bone resorption function. Expression of ASCT2 and glutaminase is induced *via* c-MYC in the early stages of differentiation, contributing to osteoclastogenesis and bone resorption function. ASCT2, alanine-serine-cysteine transporter; GLUT, glucose transporter; HIF1-α, hypoxia-inducible factor-alpha; M-CSF, macrophage colony-stimulating factor; MCT, monocarboxylate transporter; OC, osteoclast; OXPHOS, oxidative phosphorylation; PGC-1β, peroxisome proliferator-activated receptor gamma coactivator 1-β; RANK, receptor activator of nuclear factor-kappa B; RANKL, receptor activator of nuclear factor kappa-B ligand; TCA, tricarboxylic acid.

During osteoclastogenesis, there is a significant increase in glucose uptake mediated by GLUT1 and GLUT3 transporters (86). The use of inhibitors that block the glycolysis pathway or its depletion in culture media has been shown to inhibit osteoclastogenesis (86).Glycolysis also appears to be important in OC bone resorption: when mature OCs are exposed to culture a media containing only glucose, the degradation activity is markedly increased. Immunohistochemical analyses detected the presence of PKM2, and GAPDH, two glycolytic pathway-associated enzymes, near the seal zones of mature OCs, where bone resorption occurs (91).It was seen that deletion of HIF-1α and glucose deprivation inhibited the bone resorption function of OCs, along with suppression of GLUT1 and glycolytic gene expression. Accordingly, it has been proposed that HIF-1a activates the transcription of GLUT1 and glycolytic enzymes in OCs, thereby promoting glucose uptake and glycolysis, which are prerequisites for bone resorption and function (86). Therefore, during differentiation, osteoclasts exploit both OXPHOS and glycolysis to fulfill the high bioenergetic demand. On the other hand, bone resorption mainly relies on aerobic glycolysis and lactate production (**Figure 2**).

Besides glucose, amino acid metabolism plays a central involvement in regulating the OCs formation. Glutamine turns out to be critically important for OC formation. It has been shown that the hypoxic environment in which OCs are located stimulates their consumption of Gln (94). The concentration of Gln in the culture medium influences OCs formation, and its deletion inhibits both, their differentiation and function (95, 96). Following uptake through ASCT2, OCs convert Gln to glutamate and subsequently to α-KG, which is an important anaplerotic substrate in osteoclastic differentiation. The importance of the α-KG supply *via* glutaminolysis is emphasized by the fact that the inhibition of OCs formation by the deprivation of Gln is restored by the supplementation of dimethyl-α-KG (an α-KG membrane-permeable analog). In addition, the expression of ASCT2 and GLS has been suppressed by c-MYC inhibition interfering with OC differentiation and function (**Figure 2**).

Other studies have evaluated the effect of glutamate on OCs differentiation and function. Chenu’s group first and Szczesniak’s group later showed that the inhibition of NMDA receptors on isolated OCs reduced bone resorption (97, 98). In contrast to the above study, another work conducted on a mouse model of osteoclastogenesis has revealed that glutamate receptor function is not important in regulating mature osteoclast activity but is essential during osteoclast formation (99). Thus, the role of glutamate receptors in pre-OCs and OCs are not fully understood and needs further studies.

### Glutamine metabolism in the bone microenvironment in MM

Bone disease is the most frequent disease-defining clinical event in MM (1). The bone lesions result from an alteration in the normal bone remodeling which is unbalanced and uncoupled in areas of PCs infiltration. At a pathophysiological level, there is a cell-to-cell interaction between the bone microenvironment and PCs that inhibit OBs formation and enhanced OCs activity (1).

The metabolic alterations of cancer cells can alter the biochemical characteristics of the bone microenvironment and influence the metabolic behavior of microenvironmental cells. These cells adapt their metabolism to survive a hypoxic microenvironment with high concentrations of lactic acid and low levels of Gln (15). However, bone disease has been recently related to the Gln metabolism of MM cells (7, 10).

Our group has recently demonstrated that MM-imposed Gln depletion in the BM impairs OBs differentiation (7). The analysis of Gln transport in both MM and stromal cells revealed that MM cells are characterized by a higher Gln uptake than stromal cells. Consequently, MM cells rapidly consume a substantial amount of extracellular Gln depleting the medium of the amino acid. Indeed, the co-culture between MM and stromal cells decreased the levels of Gln in the medium while increasing GS expression in stromal cells. When stromal cells were differentiated in a MM-conditioned medium, they exhibit an impairment of their differentiation potential that was rescued by Gln supplementation (7). All these data suggest that MM cells can create a microenvironment characterized by lower Gln levels which, in turn, hamper osteoblast differentiation and viability. In the same way, stromal cells differentiated in the presence of a Gln concentration that mimics BM plasma of MM patients showed a significant decrease in osteoblastic markers (7). The analysis of the mechanism underlying Gln effects showed that differentiation was associated with the induction of the Gln transporter SNAT2 and the Gln-metabolizing enzyme GLS1 which were inhibited by Gln deprivation (7).

**Figure 3:**
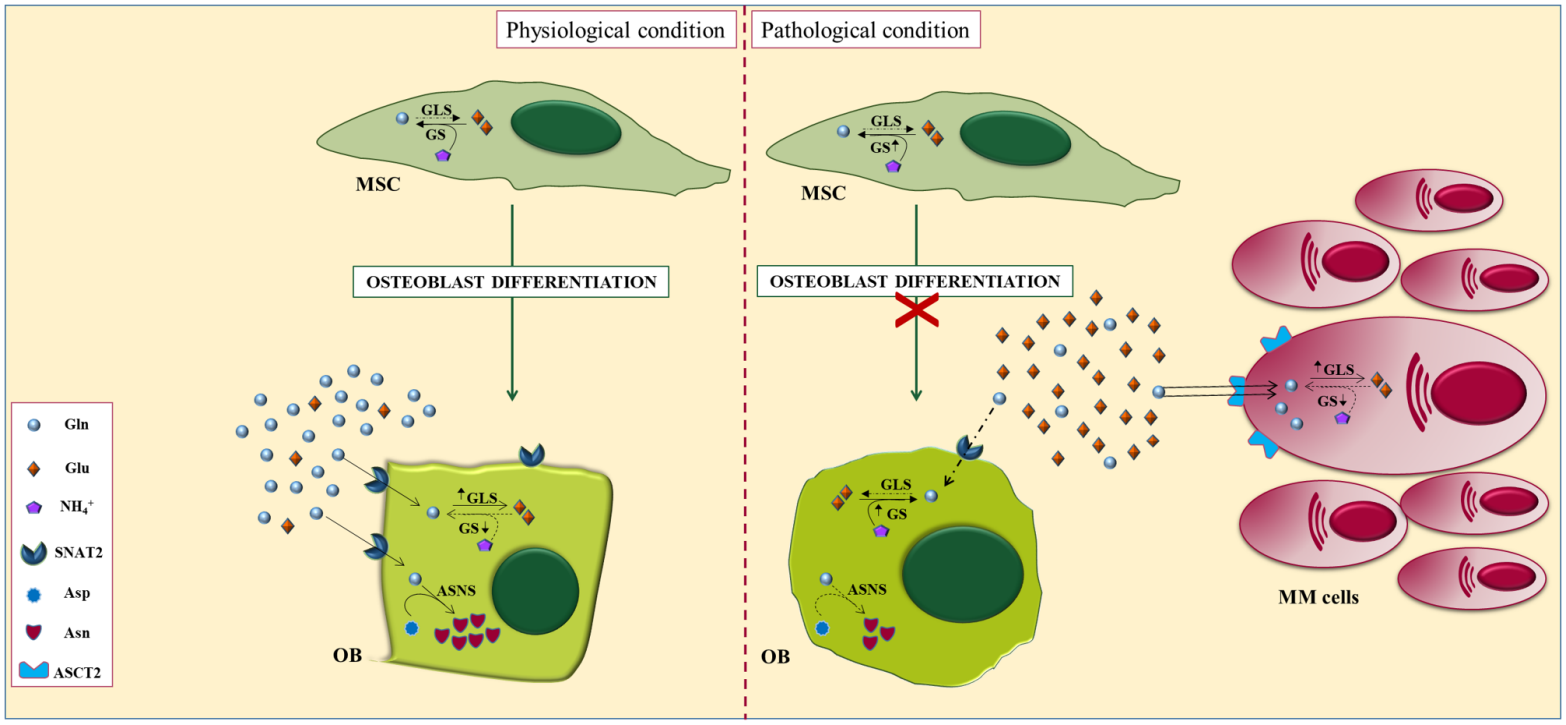
Alterations of glutamine metabolisms in myeloma bone niche. In physiological conditions (left), glutaminase and the concentric glutamine transporter SNAT2 are induced during osteoblastogenesis and are both required for the differentiation of mesenchymal stromal cells (MSCs), indicating an increased requirement for the amino acid. Osteoblastogenesis also triggers the induction of glutamine-dependent asparagine synthetase (ASNS). However, under pathological conditions (right), Gln-dependent MM cells downregulate Gln in the BM microenvironment leading to defective differentiation of OBs, attributable, at least in part, to impaired Gln-dependent Asn synthesis.

Moreover, we characterized the *in vitro* cellular content of Gln-related amino acids in differentiated MSCs. The intracellular levels of the non-essential amino acid asparagine (Asn) were found to be higher in differentiated stromal cells than in undifferentiated cells. The increase was abolished by differentiating the cells in the absence of Gln. Interestingly, the OBs differentiation impaired by Gln- deprivation was restored by Asn supplementation. The expression of asparagine synthetase (ASNS), the Gln-dependent enzyme responsible for the synthesis of Asn, increases during differentiation while its knock-out significantly attenuated the induction of osteoblastic markers in stromal cell line (7) (**Figure 3**).

Our group has recently explored the effect of Gln metabolism on OCs formation and function (10). We showed that glutamate, the main Gln metabolite, stimulates OCs formation from primary monocytes precursors isolated from premalignant patients (MGUS/SMM). In contrast, monocyte precursors from MM patients have a lower response to glutamate due to the high levels of the amino acid found in the BM (6, 10). The intracellular amino acid content analysis showed that OCs formation is characterized by increased intracellular glutamate levels. Moreover, the activity of the glutamate transporter excitatory amino acid transporter 1 (EAAT1) increases in the early step of OCs differentiation pointing to a rapid uptake of the amino acid by OCs precursors (10).

These experiments demonstrated that MM cells impair osteoblastogenesis by hindering mesenchymal asparagine synthesis through Gln depletion, providing a metabolic mechanism underlying OBs inhibition in MM. Finally, the metabolic features malignant PCs generate BM micro-environment niche characterized by low Gln and high Glu levels. Resulting in an impairment of OB formation and a stimulation of OCs differentiation. Therefore, targeting the Gln-glutamate axis could represent a novel therapeutic approach for bone disease in MM patients.

## Metabolic feature of cancer cells as a diagnostic tool: The Positron Emission Tomography/Computed Tomography (PET/CT) imaging in MM

In MM patients, bone imaging plays a central role in the detection of osteolytic lesions, as well as in staging, prognostic evaluation, and monitoring the response to therapy.

In recent years, conventional X-rays has been flanked by different radiological methods that have displayed more sensitivity in identifying the presence of osteolytic lesions and evaluating response to therapy: Whole-Body Low Dose Computed Tomography (WBLD/CT), Whole-Body Magnetic Resonance Imaging (WB-MRI), and Positron Emission Tomography/Computed Tomography (PET/CT) (100).

PET/CT is emerging as an important diagnostic and prognostic tool in the management of patients with MM. Combining morphological and functional information, PET/CT is considered the milestone of bone imaging in MM. Because MM cells have a more accelerated metabolism than normal cells, their high glucose uptake renders plausible the use of ^18^F-FDG, a glucose analog, as a PET tracer, providing very accurate information in the diagnostic evaluation of disease and beyond (101). After intravenous administration of the radiopharmaceutical drug, it is transported into MM cells, where it undergoes phosphorylation by HK2 and is converted to ^18^F-FDG-6-phosphate (6P) (102). However, ^18^F-FDG-6P is not further metabolized and becomes metabolically trapped in tumor cells. The ability of tumor cells to trap ^18^F-FDG-6P provides the basis for imaging the *in vivo* distribution of the ^18^F-FDG (102).

The analysis of metabolic disease activity by PET/CT has been shown to be relevant in the context of monitoring the response achieved with therapy in MM patients (103). The persistence of ^18^F-FDG PET/CT positivity is significantly associated with poorer survival than MM patients in whom PET/CT is negative (104). The detection by ^18^F-FDG -PET of more than three focal lesions at baseline is associated with a poor prognosis in MM patients that underwent high-dose treatment and autologous stem cell transplantation (ASCT) (103). Furthermore, the presence of at least two hypermetabolic lesions by ^18^F-FDG-PET is predictive of progression to MM patients with solitary plasmacytoma (105). In addition, in SMM patients, a positive ^18^F-FDG-PET in the absence of obvious osteolytic lesions on transmission CT may be predictive of a higher risk of progression to MM (106).

However, ^18^F-FDG-PET has some limitations related both to physiological uptake of FDG in the BM and brain and to the possible deficiency of HK2 by MM cells, which reduce its efficacy (107, 108). HK2 deficiency in MM patients has been found to cause false negativity in 10-15% of cases (108). For this reason, the use of ^18^F-FDG PET in the evaluation of minimal residual disease in low HK2 expression MM patients is not appropriate.

To overcome the limitations of ^18^F-FDG, other PET tracers have been proposed to be used in MM patients (109).

### PET/CT in MM: Beyond ^18^F-FDG

Over the past decade, molecular imaging has made significant advances. Several targets have been suggested for MM instead of ^18^F-FDG due to their increased uptake in cancer cells. Amino acid tracers are particularly interesting as biomarkers in MM cells due to their involvement in the production of immunoglobulins **Table 1**.

**Table 1:** Radiotracers explored for MM imaging discussed in this review.

Radiotracer	Physical half-life	Molecular target/Mechanism	Advantage	Disadvantage	References
**^18^F-fluorodeoxyglucose**	110 min	Glucose metabolism	Availability	False positive/negative	(101, 103, 105, 106)
**^11^C-methionine**	20 min	Amino acid metabolism	Superior sensitivity compared to ^18^F-fluorodeoxyglucose	Limited use due to on-site cyclotron need	(110–113)
**^18^F-fluoro-ethyl-tyrosine**	110 min	Amino acid metabolism	Superior sensitivity compared to CT	Data based on small sample studies	(114)
**^18^F-fluciclovine**	110 min	Amino acid metabolism	Superior sensitivity compared to ^18^F-fluorodeoxyglucose	Data based on small sample studies	(115)
**^18^F-4-fluoroglutamine**	110 min	Glutamine metabolism	Superior sensitivity in the assessment of treatment response compared to ^18^F-fluorodeoxyglucose	Data based on a preclinical *in vivo* study	(116)

The most explored amino acid tracer in MM is ^11^C-methionine as high uptake has been observed in PCs (110, 117). Several trials have compared the use of ^11^C-methionine versus ^18^F-FDG demonstrating greater sensitivity in detecting focal lesions, BM affection, extramedullary disease, and more precise measurement of tumor burden and disease activity (111, 112). The last study comparing PET with ^11^C-methionine and ^18^F-FDG PET/CT was recently published by Morales-Lozano et al. (113) in 52 patients (8 SMM, 18 newly diagnosed MM and 26 relapsed MM patients) reporting a higher sensitivity of PET with ^11^C-methionine than ^18^F-FDG (113).^11^C-methionine detected tumor infiltration in 11% of ^18^F-FDG PET/CT negative MM patients, detecting a greater number of lesions in the majority of patients. Additionally, ^11^C-methionine showed correlation with BM infiltration, it could suggest this tracer better reflects the tumor burden than ^18^F-FDG (113).

The ^18^F-fluoro-ethyl-tyrosine (^18^F-FET) is a tracer already used in the diagnosis of brain neoplasms (118). The ^18^F-FET is uptake and incorporated into newly synthesized proteins, such as ^11^C-methionine (117). In a preliminary study, the use of ^18^F-FET proved to be more sensitive than low-dose CT by detecting 83 lesions vs 64 in patients with newly diagnosed MM. Two patients had more lesions with ^18^F-FET while in six the number of lesions detected using CT and ^18^F-FET was the same. Patients in complete remission from the disease had no FET-positive lesions (114).

An additional amino acid tracer considered for MM PET imaging is ^18^F-fluciclovine. ^18^F-fluciclovine is a radiolabeled analog of leucine, which is an essential amino acid (119). Like leucine, ^18^F-fluciclovine is uptake through the amino acid transporter systems, demonstrating maximum uptake in tissues that produce proteins or process amino acids (120). In a prospective study, thirteen patients with MM before and after first-line treatment with induction therapy and ASCT underwent both ^18^F-fluciclovine and ^18^F-FDG PET/CT. Compared with ^18^F-FDG PET, ^18^F-fluciclovine showed significantly higher uptake, detecting more lesions in seven of the thirteen patients. Moreover, three patients who were negative for ^18^F-FDG PET were positive for ^18^F-fluciclovine PET/CT (115). All ^18^F-fluciclovine-positive PET/CT MM patients exhibited a satisfactory response to treatment three months post-ASCT. Interestingly, a correlation between the uptake of ^18^F-fluciclovine PET and the percentage of neoplastic PCs in BM biopsies was found, which was not observed with ^18^F-FDG (115).

In summary, emerging evidence supports the promising role of these amino acid tracers in the management of MM patients. Preliminary reports are mostly encouraging, indicating a more sensitivity in particular settings. However, the clinical impact of these new tracers is not yet known due to the limited number of patient cohorts studied. Further studies are needed to better ascertain the clinical settings in which these tracers will provide an added value and definitive clinical impact.

### ^18^F-4-Fluoroglutamine as a possible new tracer in MM

Glutamine dependence has long been reported as a metabolic feature of several malignancies, suggesting the possible use of its radiolabeled analog, ^18^F-4-fluoroglutamine (FGln), as an alternative PET tracer. Several papers evaluating clinical safety, pharmacokinetics, and imaging have been published in support of its possible use as an imaging biomarker (121–123). The possible exploitation of Gln as a PET tracer has been successfully evaluated in patients with lymphoma (124, 125). Recently, Valtorta et al. (116) evaluated the uptake of ^18^F-4-FGln in MM mouse models in comparison to ^18^F-FDG (116). Both radiotracers identified MM cells colonization *in vivo*. Mice were treated with bortezomib and underwent PET scan with both radiotracers. Based on the tumor volume in response to bortezomib treatment, mice were classified as responder and non-responder. Responder mice showed a reduction of ^18^F-4-FGln uptake while the ^18^F-FDG increased in both groups, showing a higher affinity of ^18^F-4-FGln to the sensitivity of the drug (116). Overall, these data indicate that ^18^F-4-FGln could be useful as alternative PET tracer and to describe the alterations induced by therapy. The characterization of the metabolic profile of malignant PCs could be exploit to design novel metabolic therapeutic approaches.

## Conclusion

Growing evidence reviewed herein suggest that the metabolic rewiring of MM cells creates a peculiar BM niche with low glutamine and high glutamate levels. The metabolic reprogramming of the microenvironment affects both OBs and OCs differentiation leading to alterations of bone remodeling process contributing to the development of osteolytic bone disease. Based on this data, new treatments with metabolism-altering agents emerged from preclinical evidence could be explored in the future in a clinical perspective in MM patients.

Moreover, the alterations of metabolic features of MM cells and their bone microenvironment may represent a new diagnostic tool in MM patients. In this context, we have underlined the possible use of ^18^F-4-FGln as a novel PET tracer showing encouraging results in MM tumor burden detection and in the evaluation of treatment response in preclinical *in vivo* models. Based on these data, we can argue that ^18^F-4-FGln PET could help to better define the metabolic phenotype of the tumor and the changes induced by therapy. Finally, *in vivo* study of the metabolic profile of myeloma cells by ^18^F-4-FGln could be useful for designing future metabolism-based therapeutic approaches and for the clinical management of MM patients.

## Author contributions

VR, DT, and JB-G wrote the manuscript; VM and VR designed the figures. All authors commented on previous versions of the manuscript. All authors read and approved the final manuscript.

## Funding

This work was supported by a grant from “Associazione Italiana per la Ricerca sul Cancro” (AIRC) under IG2017 ID. 20299 and International Myeloma Society (IMS) under “Paula and Rodger Riney Foundation Translational Research Grant”.

## Conflict of interest

The authors declare that the research was conducted in the absence of any commercial or financial relationships that could be construed as a potential conflict of interest.

## Publisher’s note

All claims expressed in this article are solely those of the authors and do not necessarily represent those of their affiliated organizations, or those of the publisher, the editors and the reviewers. Any product that may be evaluated in this article, or claim that may be made by its manufacturer, is not guaranteed or endorsed by the publisher.
